# The Production of Complement Inhibitor Proteins in Mammalian Cell Lines—Light at the End of the Tunnel?

**DOI:** 10.3390/biomedicines12030646

**Published:** 2024-03-14

**Authors:** Attila Szvetnik, Vilmos Tubak

**Affiliations:** Creative Laboratory Ltd., Kereskedő köz 5/C, 6728 Szeged, Hungary; vilmos.tubak@creativelab.hu

**Keywords:** complement, complement inhibitor, factor H, CR1, recombinant protein expression, mammalian cell lines, stabilon, codon usage, mRNA optimization

## Abstract

Therapeutic recombinant proteins are powerful tools used for the treatment of many detrimental diseases such as diabetes, cancer, multiple sclerosis, rheumatoid arthritis, hepatitis, and many more. Their importance in disease therapy is growing over small molecule drugs because of their advantages like specificity and reduced side effects. However, the large-scale production of certain recombinant proteins is still challenging despite impressive advancements in biomanufacturing. The complement cascade is considered a rich source of drug targets and natural regulator proteins with great therapeutic potential. However, the versatility of such proteins has been hampered by low production rates. The recent discoveries highlighted here may bring definite improvement in the large-scale recombinant production of complement inhibitor proteins or other difficult-to-express proteins in mammalian cell lines.

## 1. Introduction

The growing number of diseases, pathogenetic states, and clinical conditions associated with complement dysfunction fuel a strong interest in therapeutic options to modulate this host defense pathway. Among the various types of drug candidates, natural complement inhibitor proteins and derived molecules seem to be outshined by novel innovations in the current boom [1,2]. However, particularly in diseases in which a complement factor function is missing (lacking or mutated; for, e.g., factor H and aHUS), there is hardly any other option than replacement. A factor purified from blood cannot meet the scale of demand; thus, recombinant production is basically the only and highly desirable option [3]. Several companies and academic groups are encouraged to meet this demand with factor H or CR1-based molecules.

Factor H is the most abundant and one of the most important complement inhibitors. FH mutations are associated with severe human renal and retinal diseases [4], including atypical hemolytic uremic syndrome (aHUS) [5], membranoproliferative glomerulonephritis II (MPGN II) [6,7], or age-related macular degeneration (AMD) [8]. It is not surprising that most of the inhibitor proteins currently being considered drug candidates are related to factor H. Small derivatives such as AMY-201 (mini-FH; Amyndas Pharmaceuticals), MFHR1, FHL-1, and full factor H (Compleva MFHR1, -FHL, and -FH; eleva GmbH, Freiburg, Germany) are under research or undergoing a preclinical phase of drug development. The abundance of FH in plasma suggests a presumably high therapeutic dose, similar to or even higher than the 5–20 mg/kg utilized in mAb therapies [9], not considering protein stability issues or frequency of administration.

A different type, membrane targetable molecule (Mirococept or APT070) derived from CR1 [10,11,12], is under investigation at King’s College London for preventing ischemia-reperfusion injury associated with kidney transplantation. A truncated soluble variant of CR1 lacking the LHR-D domain (CSL040; CSL Ltd., Melbourne, Australia) has recently been introduced [13] and characterized by exhibiting greater in vitro potency and an extended in vivo half-life compared with full-length CR1. A Phase I clinical study with this molecule will start soon in Australia.

**Figure 1:**
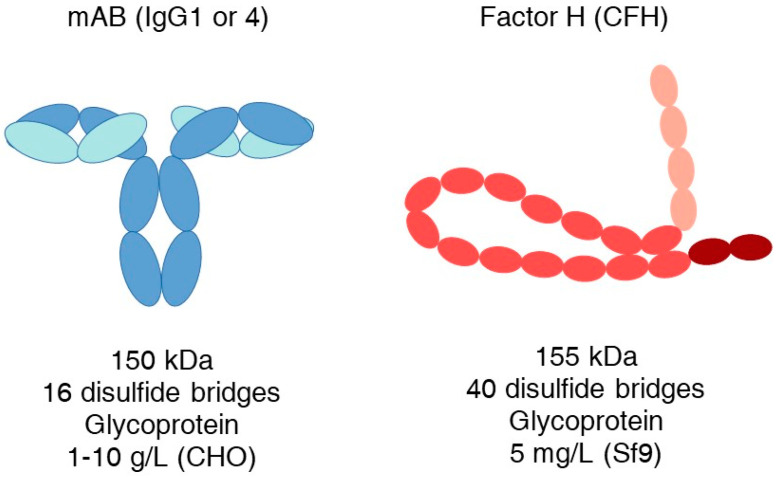
Comparison of some characteristics and highest production rates of a mAb and human factor H. Heavy chain—darker blue; light chain—cyan; pink SCRs are the C3b binding region; brown SCRs form a surface attachment region.

Efficient recombinant production on a large scale is crucial for the success of the above-mentioned and similar future drug candidates. Despite the considerable efforts over the past three decades, the production of complement inhibitory proteins has remained difficult and hinders therapeutic applications. It is a startling contrast, however, that antibodies similar in overall size to, for example, factor H, possess complex folding and disulfide bridges and can still be produced in CHO cell lines at levels of 1–10 g/L (Figure 1).

Here, we provide an overview of published complement regulator protein production rates in various eukaryotic expression systems investigated so far and highlight some recent technical developments in the field of recombinant protein expression that may enable bridging the efficiency gap between the production of mAbs and complement inhibitor proteins in eukaryotic host organisms.

## 2. The Therapeutic Value and Market for Complement Inhibitors Is Rapidly Expanding

The first complement inhibitory drug, the C5-specific monoclonal antibody eculizumab (SOLIRIS, Alexion Pharmaceuticals), was approved in 2007 for the treatment of the rare disease paroxysomal nocturnal hemoglobinuria (PNH). Since then, eculizumab has also been used to treat atypical hemolytic uremic syndrome (aHUS), generalized myasthenia gravis (gMG), and neuromyelitis optica spectrum disorder. Before the introduction of eculizumab, the prognosis for these diseases was poor. The 10-year mortality of PNH was approximately 50% [14]; about 50% of patients suffering from aHUS died or acquired end-stage renal disease within one year of diagnosis [15], and the mortality was 67% of adults within 5 years [16]. In contrast, eculizumab treatment rapidly improves and maintains a stable kidney function for the long term in aHUS [17,18], and a five-year survival rate of 98.3% has been reported in PNH patients with treatment [19].

The therapeutic and commercial success of eculizumab initiated a rapid increase in interest in the field. A major shift has taken place as the complement system has been recognized as a key pathological contributor and therapeutic target [20,21]. At present, twelve inhibitors, including C1-INH, pegcetacoplan, and avacoplan, are approved by the FDA, and >50 other late-stage drug candidates are in development [2,3,22].

There are several factors driving the current ”clinical complement revolution”. First, available treatments have dramatically improved patients’ quality of life and survival expectancy, but at a great cost. Complement inhibitor drugs (Soliris, Ultomiris, Cinryze) are among the most expensive pharmaceuticals in the world. The annual cost can exceed USD 4–600,000, which is a serious financial burden for patients. Therefore, there is a huge need for alternative and effective but more reasonable medications.

Secondly, the complement system’s role and pathological contribution to various diseases and clinical conditions is becoming more and more recognized. Besides the complementopathies in which the disorder is driven by complement, there are many more medical states where complement significantly or substantially contributes to the condition. A large portion of patients is affected by complement dysfunction in cancer, diabetes, AMD, pre-eclampsia, sepsis, chronic inflammation, different autoimmune diseases, ALS, cold agglutinin disease (CAD), multiple lung diseases (e.g., ARDS, COPD, and pneumonia reviewed in [23]), COVID-19, schizophrenia, Alzheimer’s disease, rheumatoid arthritis, IBS, ischemia-reperfusion, and during organ transplantation [3,24,25,26].

In addition, severe, sometimes fatal complement overactivation has long been observed during the systemic administration of drugs. Such infusion-related hypersensitivity reactions or complement activation-related pseudo allergy (CARPA) can be caused by nanomedicinal particles, mAbs or antibody fragments, liposome-based formulations (e.g., Doxil and RNA vaccines), enzymes, and adenovirus-based drugs (gene therapy) [27,28,29].

Significant complement activation occurs when blood is drained from the circulatory system, for example, during hemodialysis (>550,000 patients in the USA), hemofiltration, plasmapheresis, plasma exchange, apheresis, extracorporeal membrane oxygenation (ECMO), cardiopulmonary bypass (CPB), and the use of an extracorporeal ventricular assist device [30,31,32,33,34,35,36].

In many cases, the co-administration of complement inhibitors with therapeutic agents or procedures could reduce the risk of potential hyperactivation. Natural inhibitors such as factor H or sCR1 and derived proteins possessing short half-lives [37,38,39] seem ideal for mitigating transient complement overactivation.

Finally, complement biology is a rapidly evolving and growing field of research that is constantly yielding exciting, novel discoveries. For example, the role of neuronal sushi domain protein (SRPX2) as a soluble complement inhibitor in the central nervous system was described relatively recently [40]. It regulates complement-mediated synapse elimination in the brain, which plays an important role in neurodevelopment and neurological diseases. Moreover, the importance of complement activity in the gut and breast milk in the selective clearing of pathogens has just been described [41,42].

**Figure 2:**
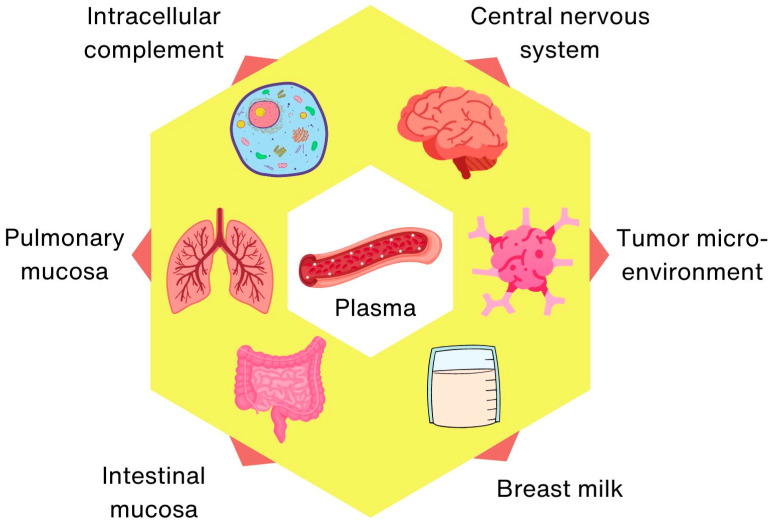
Increasing functional complexity of the complement system.

The activity of the complement system has traditionally been attributed to its presence in the circulatory system, but on the basis of accumulated knowledge [43,44,45], separate intracellular [46,47,48], lung [23] and gut (mucosal) [34], neuronal [49,50], tumor microenvironment [51] and breast milk [35,52,53] complement systems can be defined, characterized by the local production of (sometimes non-canonical) components and unique functions (Figure 2).

This explosion of knowledge has led to the identification of new drug targets and inhibitor candidates, boosting research funding and also driving investment by Big Pharma. According to market analysts, the complement inhibitor retail is expected to continuously grow by more than 15% annually from an estimated USD 6.8 billion (2022). More than half of this revenue comes from the sale of mAbs (Soliris and Ultomiris), and the importance of mAbs in future complement therapy seems evident. Eculizumab (Soliris) is produced in the NS0 murine myeloma cell line, while ravulizumab (Ultomiris) is produced in Chinese hamster ovary (CHO) cells. This not only underlines the importance of protein production in mammalian cell lines but also shows the great clinical and commercial potential of natural complement inhibitors in case of efficient production.

## 3. Non-Mammalian Hosts Are Ahead

Complement regulators have been expressed in bacterial, yeast, plant, insect, and mammalian cells as well. Although a great number of papers reported on the purification of such proteins, in most cases, the actual yield is not presented. Bacterial expression is not considered here because of a lack of glycosylation, the complex downstream process, including the in vitro refolding of the insoluble protein produced, and the removal of endotoxin [54,55], which makes large-scale production less competitive.

Generally, the preferred system to manufacture protein-based therapeutics is mammalian cell expression, due to the requirement of post-translational modifications (PTMs) for activity. CHO cell line is the most commonly used mammalian system and is the manufacturing host for >70% of approved biotherapeutics [56]. It is a robust system capable of high protein productivity in suspension culture and propagates well in a serum-free medium. CHO cells confer glycosylation features to glycoprotein products; nevertheless, they generate non-completely human glycan structures, which carry an increased risk of immunogenicity. When PTM is crucial, HEK293 cells are the primary choice as they offer multiple advantages in addition to the ability to generate human glycosylation profiles. However, both CHO, HEK293, and other mammalian cells proved to be quite inefficient in manufacturing complement inhibitor proteins (Table 1). The insect *Spodoptera frugiperda* Sf9 cell/baculovirus expression system was the most efficient non-microbial producer with 5 mg/L full-length factor H and up to 9 mg/L for shorter molecules. However, the expensive media, the non-human glycosylation, and the inability of continuous production due to cell lysis upon viral infection make it less attractive for large-scale production [57].

Due to several advantages, such as growth in simple, inexpensive media, low toxicity risk, scalable production with good yields, and product secretion, the methylotroph yeast *Pichia pastoris* has become the host organism of choice. Proteins produced in *Pichia* had a tremendous impact on the complement field, and a great body of knowledge has been collected by this versatile expression system [58,59,60]. Currently, the highest production rate of the human factor H (15 mg/L) in *P. pastoris* represents a major advancement and an opportunity for further development (see more details in Section 7).

Another promising approach that has recently been significantly developed and optimized is bryotechnology [61], which utilizes bryophyte cells for protein production. The expression system of the moss *Physcomitrella patens* enables the cost-effective and pathogen-free production of a variety of biopharmaceuticals, for example, the moss-aGal [62]. Via genetic engineering, the full human glycosylation pattern has been achieved, and a standardized photobioreactor cultivation process with a scale up to 500 L is feasible [61,63]. Using this technology, eleva GmbH (Freiburg, Germany) has been engaged in the development of FH, MFHR1, and FHL-1 complement regulators.

**Table 1:** Examples of complement inhibitor production yields in eukaryotic hosts.

Protein	Host	Production	Citation
CFH	Sf9	5 mg/L	[64]
CFH	*Pichia pastoris*	3–5 mg/L	[65]
CFH	COS-7	0.2–2 mg/L	[66]
CFH humanized	*Physcomitrium patens*	<1 mg/L	[63]
CFH	*Physcomitrium patens*	0.0258 mg/g DW/5 days ^1^	[67]
CFH	*Pichia pastoris*	~15 mg/L	[68]
rat FH(SCR1–4)	HEK293	~0.6 mg/L	[69]
mini-FH (FH^1–5^18–20^)	CHO	2–4 mg/L	[37]
mini-FH (FH^1–5^18–20^)	*Pichia pastoris*	Not reported	[70]
mini-FH (FH^1–5^18–20^)	Sf9	8.2 mg/L	[71]
FHL-1	Sf9	9 mg/L	[72]
MFHR1 MFHR13	*Physcomitrium patens*	0.1 mg/g FW 0.170 mg/g FW ^2^ 1.4 mg/L/8 days	[73,74]
CR1(SCR15–17) CR1(SCR15–16) CR1(SCR16)	*Pichia pastoris*	30–100 mg/L	[75]
CR1(SCR1–3)	CHO	1 mg/L	[76]
CSL040(SCR1–21)	CHO	Not reported	[13]
mDAF1 mDAF11	*Pichia pastoris*	15–20 mg/L	[77]
rat CD59-Crry rat Crry	*Pichia pastoris*	10–100 mg/L	[78]
MCP	COS-7	0.4 mg/L	[79]

^1^ According to the 1 g/L biomass dry weight published, the current density is 3–4 g/L dry weight (DW; from the website of leva GmbH). ^2^ FW—fresh weight.

Based on the observed production efficiencies (Table 1), complement inhibitors composed of SCRs (short consensus repeat domains) can be characterized as weakly expressible proteins in all production systems. The modest production of a few mg/L in mammalian cell lines is about two orders of magnitude lower compared to highly ex-pressed bioproducts such as monoclonal antibodies. However, this dramatic difference suggests that there is plenty of room for further development.

## 4. The General Strategy of Recombinant Protein Expression in Mammalian Cells

**Figure 3:**
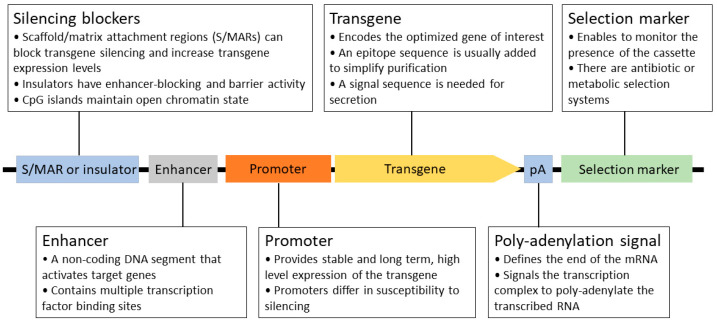
Schematic structure of a stably integrated mammalian expression cassette.

**Figure 4:**
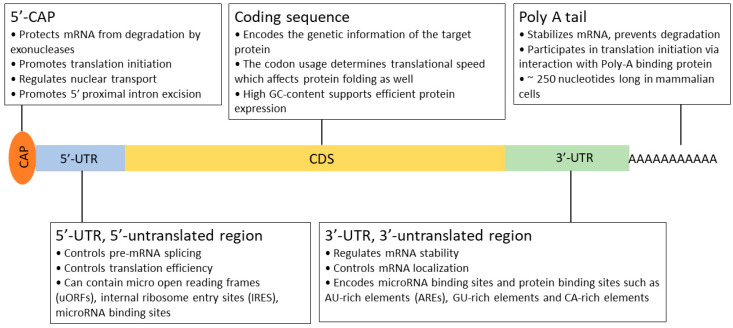
Schematic structure of the functional elements of a mature eukaryotic mRNA.

Recombinant protein expression is a complicated, multistep process that requires significant development time. In order to achieve the current level of production and quality, many key aspects have been defined and optimized over the past 30 years. The generation of stable cell lines is essential for large-scale protein production. In this process, a well-designed expression construct is randomly or site-specifically integrated into the chromosomal DNA of the host. As a rule of thumb, high mRNA levels are required for efficient protein production [40,41]. Therefore, all constituents of the expression system serve this goal, from the choice of vector, the composition of the expression cassette (promoter, 5′- and 3′ UTRs; Figure 3 and Figure 4), the optimization of the transgene (secretion signal, codon preference, and purification tag), and mode of integration and selection to elements preventing the possible negative position effects and silencing (e.g., S/MARs) after integration [80]. Usually, a higher transgene copy number results in a higher final protein titer, but it can also lead to genomic instability. After a selection period, individual colonies are picked and characterized by their protein production, stability, and growth properties.

The secretion of recombinant protein into the culture medium during industrial-scale protein expression has many advantages for downstream processing. The supernatant contains significantly fewer contaminating proteins and other metabolites, thus allowing for a straightforward and more effective protein purification process. In this way, the producing cells can also be maintained for longer with continuous feeding. Batch cultures typically achieve less than 5 × 10^6^ cells/mL cell density and 0.5 g/L product concentration [81]. In contrast, fed-batch and continuous culture systems allow for a high >10 × 10^6^ cells/mL cell density and 1–10 g/L product titer [81,82,83]. 

The amount of protein produced in stable secreting cell lines can vary dramatically for different target proteins, and the reasons for this are not well understood and are difficult to predict [84]. Each step of the process must be reasonably efficient and streamlined to achieve active protein secretion: transcription, translocation of mRNA to the ER surface, an appropriate translation rate, proper folding in the ER, glycosylation, and finally, secretion from the cell. Oversaturation of the system at any step can lead to cell stress, slow growth, decreasing protein titer, or apoptosis.

Optimizing protein production is a painstaking, multistep investigation. However, we may already have the right tools to fine-tune the individual layers.

## 5. The Emerging Toolbox of Expression Controlling Short Motifs

There are various ways to facilitate protein expression from the transgene within the cell. Since the mRNA levels correlate with protein production, the half-life of mRNA is a crucial factor, but the subsequent translational rate and efficient secretion are key features as well. Enhancement of these steps has now been demonstrated by the simple incorporation of short RNA motifs.

### 5.1. The SECReTE Motif

Numerous experimental data suggest that mRNA targeting to the ER is not restricted to the signal recognition particle pathway but likely involves different paths as well. Cohen-Zontag and coworkers [85] performed a detailed bioinformatic analysis in the budding yeast transcriptome to reveal specific mRNA-sequence motifs in secreted proteins. Finally, short NNY repeats were identified that are overrepresented in mRNAs encoding secretome proteins. These stretches, termed SECReTEs, consist of pyrimidine repeats at every third base (*NNY*, *N =* any nucleotide, *Y* = C or U) that can be ≥10 nucleotide triplets in length and can reside both in the coding sequence and 5′- and 3′-UTRs in yeast. The authors found the SECReTE motif in other yeast, bacterial, and human transcriptomes, indicating a conserved evolutionary role. The physiological relevance of SECReTE was explored by abolishing or adding motifs to three yeast proteins (*HSP150*, *SUC2*, and *CCW12*) and artificial GFP reporter constructs. The experiments proved that the number of SECReTE motifs not only increased protein production and secretion and stabilized or even enhanced the mRNA levels but also promoted the localization of transcripts to the ER. 

Thus, a novel cis-acting RNA motif has been described in yeast that facilitates ER-localized mRNA translation and protein secretion, which may have great biotechnological relevance in industrial protein expression. In contrast to yeast, the human SECReTE motifs are preferentially located in the 5′-and 3′-UTRs, and their physiological significance needs to be confirmed in mammalian cell lines.

### 5.2. RNA Hairpin Regulation Elements (RgEs)

It was previously known [86] that protein expression in mammalian cells is dependent on thermodynamic stability, GC content, and the relative position of RNA hairpins at the 5′-UTR. To intentionally and predictably control protein expression levels in mammalian cells in support of recombinant protein production, Eisenhut and co-workers [87] constructed a panel of defined RNA hairpins termed “Regulation element(s)” or short “RgE(s)” and characterized them for their ability to tune protein expression in two of the most commonly used mammalian expression hosts, CHO-K1 and HEK293 cells. Messenger RNA levels of fluorescent reporters (RFP and BFP) were determined in transient expression experiments to confirm that the RgEs influence the translation process.

The designed 25 RgEs regulated protein expression levels from 110% to almost 0% of the initial promoter activity. As demonstrated, RgEs functioned in a comparable manner in cell lines of different mammalian organisms, and stable hairpins mediated strong repression, whereas weak hairpins reduced expression levels less strongly or even enhanced them. The presence of RgEs affected reporter mRNA levels, calculating the RNA to protein fold change ratio revealed that instead of RNA, protein expression level was the most important influencing factor.

Possible use of RgEs was demonstrated in the production of trastuzumab as a model system in the transient expression of ExpiCHO™ cells. Several RgEs were cloned into the 5′-UTR of the heavy chain gene to control the relative expression of HC. Interestingly, several RgE constructs with reduced HC expression dramatically increased mAb titer, with a maximum of 3.5-fold yield. Furthermore, the enhanced production was accompanied by much better quality, as evidenced by the increased signals for full-size mAb and HC-LC fragments in the samples.

In consequence, RgEs appear to be excellent tools to systematically tune protein expression in mammalian cells and help to optimize recombinant protein expression, especially those that are difficult to express or composed of multiple subunits. 

### 5.3. Short RNA and Protein Stabilizers

The concept of a short peptide tag with the ability to stabilize fusion partners is not new. Such a sequence was identified in the C-terminus of the transcription factor DP-1 [88]. This negatively charged, 16-amino-acid-long peptide (and its shorter derivatives) not only affected the stability of DP-1 but also dramatically increased the GAL4DBD protein cellular levels when fused to its C-terminus in HEK293 cells. Unfortunately, the mechanism of the DP-1 STABILON remained unresolved, and no further information could be found.

Recently, a similar short (13 aa), highly charged protein motif (KDGKKDKKEEDKK) called STABILON was isolated from the p54/Rpn10 ubiquitin receptor subunit of the *Drosophila* 26S proteasome [89]. It was shown to strongly increase the expression levels of various intrinsically unstructured proteins (c-Myc-Stab, ERD-10-Stab, and Gly-H-Stab) in CHO-K1. Moreover, a C-terminally fused STABILON on human erythropoietin (hEPO) dramatically elevated not only the intracellular but also the secreted and fully glycosylated form of the protein. Analysis of transcript levels by quantitative RT-PCR revealed that mRNA levels of STABILON-fused transgenes were 4–15-fold higher than those of parental transgenes. Based on the experimental results, the STABILON motif can provide increased mRNA levels of the transgenic fusion products and also stabilizes proteasomal degradation-prone proteins in *Drosophila melanogaster* and CHO-K1 cell cultures. According to the authors, STABILON represents a genuine *cis*-acting mRNA and protein stabilization signal.

Most recently, another mRNA stabilizing cis-regulatory motif called Exin21 was reported [90]. The presence of this short, 21-nucleotide-long segment (CAACCGCGGTTCGCGGCCGCT) dramatically increased the expression and secretion of several difficult-to-express proteins such as SARS-COVID2 envelope or Spike proteins and a mAb in human cell lines. Importantly, the authors observed an average 13-fold (maximum of 37-fold) increase in the tested human anti-SARS-CoV mAb concentration secreted into the medium. This specific sequence in the particular order is necessary for its beneficial effects, as demonstrated via alanin scanning, induced deletions, and synonymous and non-synonymous mutations. Taken together, Exin21 fused to a target mRNA can significantly increase nascent mRNA synthesis and mRNA stability of the target mRNA, increase protein expression, and also facilitate secretion of the target protein.

**Figure 5:**
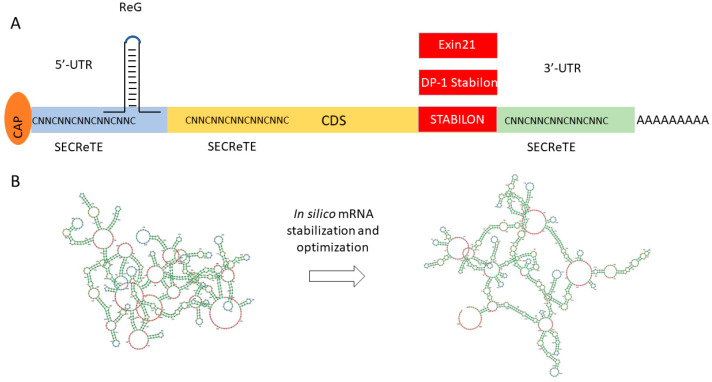
Novel tools for mRNA stabilization and expression optimization. (**A**) Expression controlling short motifs. The preferred location of the motifs ReG, SECReTE, Exin21, DP-1 Stabilon, and STABILON are shown in the figure; red rectangles show C-terminal translational fusion. (**B**) Complex mRNA sequence optimization of FH(SCR1–4) using mRNAid (https://mrnaid.dichlab.org/ (accessed on 15 December 2023)).

In summary, a variety of easily applicable sequence motifs (Figure 5A) are available today that have been demonstrated to enhance mRNA and/or protein stability and even boost target protein secretion. Although the exact mode of action is largely unknown, their great potential for industrial protein production seems obvious.

## 6. Complex Fine Tuning of RNA Coding Sequence Instead of “Codon Optimization”

All different organisms and species have a consistent preferred usage of synonymous codons, which is called codon usage bias. Thus, when a protein of interest is expressed in a heterologous system, the transgene should be adapted to the codon usage bias of the new host. In this way, the risk of depletion of the tRNA pool is minimized, which should result in a faster translation rate and high recombinant protein levels.

So, all that is needed is to exchange all the rare codons for optimal codons in the transgene, and we can harvest grams of proteins. Unfortunately, the case is much more complicated than that. In practice, such a simple codon optimization often leads to unpredictable consequences [91,92,93,94]. Even a single synonymous mutation can significantly alter the formation of messenger ribonucleoprotein particles (mRNPs), mRNA secondary structure, mRNA stability, microRNA binding, translation, and protein folding [95,96,97]. Hence, elimination of all the additional layers of information encoded in the mRNA sequence should be avoided.

For instance, the time required for the proper folding of each domain in a protein can vary greatly. The folding process can be hindered by an excessively rapid translation rate, which can result in aggregation or an inactive product. The above-mentioned cautionary example of RgE-trastuzumab [87] highlights this, as a greatly decreased (to ~35%) translational rate of HC provided the highest mAb titer and better quality as well.

Other observations also suggest that there is a “sweet spot” of ribosome loading and translation efficiency to achieve maximal total protein output. Mathias et al. [98] investigated the problem of difficult-to-express antibodies by following the faith of an underperforming mAb in CHO cells. The experiments suggested that transcription and translation were as efficient as in a good mAb producer cell line; even so, the problematic mAb accumulated in the ER, causing aberrant ER morphology and mainly failing to secrete. Further experiments clearly revealed that the antibody’s light chain (LC) was only partially folded, and disulfide bridges had not been properly formed. During the maturation of antibodies, the heavy chain constant domain 1 (CH1) only folds in the presence of an already folded LC; thus, the whole antibody folding was poised, which led to mAb aggregation in the ER. 

It has been extensively shown that codon optimality of a transcript strongly correlates with mRNA half-lives in many eukaryotic species, including humans [99,100]. In fact, the mRNA degradation complex (CCR4/NOT) senses ribosome translocation rates, and slow translation triggers the deadenylation of the polyA-tail and mRNA decapping (COMD; [101]). However, sequence content dramatically impacts mRNA half-lives; codon optimality is only one of many principles guiding mRNA stability. COMD impact on mRNA half-lives varies between and within species: in budding yeast, COMD accounts for 60% of all mRNA half-lives [99,102], while in human cell culture, the impact is much lower [100,103].

Leppek and co-workers [104] simultaneously delineated the in-cell mRNA stability and ribosome load for a combinatorial library of 233 Nanoluciferase mRNAs in HEK293 cells. The developed PERSIST-seq technology enables parallel evaluation of the effects of UTR, CDS sequence, and structure on in-cell mRNA translation efficiency, in-cell mRNA stability, and in-solution stability. Interestingly, the maximal protein output was not produced from the mRNAs with the highest translational efficiency (heaviest polysomes). According to the authors’ opinion, this effect may derive from overcrowding of ribosomes on mRNA coding regions due to efficient and rapid translation initiation, which can induce ribosome queuing and sterical ribosome collisions, which have recently been found to lead to translation-dependent mRNA decay. Therefore, mRNAs seem to be more stable with an optimal number of ribosomes loaded for efficient protein synthesis. 

Another key observation was the importance of mRNA secondary structure for efficient protein expression. By reducing the overall presence of Us in loops and reducing the number of hairpins to generate a “linear” highly double-stranded mRNA (“superfolder”) could be formed; in consequence, mRNA stability and protein expression were enhanced.

Similarly, mRNA codon optimization and simultaneous structural stabilization have been recently performed by the algorithm LinearDesign [105]. The authors used the classical concept of lattice parsing in computational linguistics for the optimization process, which strikingly reduced computational time. In line with the previous results [104], the stabilization of SARS-COVID2 Spike mRNA enabled 2.3–2.9-fold higher protein expression and a higher immunogenicity as well. For this, only the coding sequence was used as an input; thus, LinearDesign is likely to remain effective independent of the choice of UTRs.

Therefore, codon optimality is not the only principle guiding in-cell mRNA stability, and optimal secondary structure may be a greater driver of protein output. Rather, there is an interplay with other RNA features such as GC content, structure, miRNAs, and binding proteins to dictate mRNA fate. Also, the translational elongation rate is influenced by other factors as well, such as amino acid availability, polypeptide composition, RNA binding proteins, cellular stress, or the dynamically changing tRNA pool [59].

There are a lot of available codon optimization tools. However, a combination of multiple optimization strategies is complicated and computationally expensive. This was attempted by Vostrosablin and coworkers [106] mainly for the rational design of mRNA vaccines. The created open-source software mRNAid (version 1.0.0) is for mRNA optimization and visualization that generates mRNA sequences with superior characteristics. It orchestrates the simultaneous optimization of several sequence and structural properties, including codon usage, GC content, minimum free energy (MFE), uridine depletion, and exclusion of specific motifs and/or rare codons. The experimental validation was performed by a NanoLuciferase-PEST (Nluc-PEST) reporter system, and similarly to LinearDesign, a 2–3-fold protein expression increase could be achieved after optimization. An easy-to-use, online accessible version of mRNAid is available at https://mrnaid.dichlab.org/ (accessed on 15 December 2023).

## 7. Enhancing Protein Folding by Protein Disulfide Isomerase Co-Expression

A promising or even game-changer result has been published regarding FH production in *Pichia pastoris* [68]. Since FH contains 40 disulfide bridges, the authors hypothesized that limited levels of protein disulfide isomerase (PDI) activity within the endoplasmic reticulum (ER) might be a key limiting factor during production. PDI accelerates the formation of disulfide bridges and the folding process as a chaperone. Correctly folded proteins in the ER are subsequently transferred to the Golgi prior to secretion, but the insufficiency of PDI may occur, leading to the accumulation of misfolded conformations with non-native disulfides. These activate quality-control pathways of the unfolded protein response (UPR), including increased chaperonin production, translation arrest, and ER-associated protein degradation. 

To circumvent possible folding limitations, the PDI was co-expressed with a codon-optimized murine FH, and indeed, the yield strikingly increased more than 100-fold compared to yeast cells not producing extra PDI. The same strategy was applied for hFH production, resulting in the highest published yield so far (~15 mg/L; Table 1) with any expression system or host.

A similar bottleneck may very well restrain the production in mammalian cell lines, especially during highly overexpressing conditions optimized for proteins that contain only a few disulfide bridges per molecule. Therefore, the application of PDI co-expressing mammalian cell lines appears to be an important additional tool for reaching efficient complement inhibitor production sufficient for therapeutic purposes.

## 8. Conclusions

In this review, we highlighted some novel tools that have remarkable innovation potential in the production of difficult or weakly expressed proteins, such as complement inhibitors in mammalian cell lines. It will take a lot of further effort to explore the useful combinations of the described available RNA or protein stabilizer short motifs, rational design of coding sequence, and utilization of host cell lines co-expressing protein disulfide isomerase and implement them with already well-known critical factors for efficient protein expression. However, the high-level production of full-length complement inhibitors or shorter derivatives may be closer than previously anticipated.

## Data Availability

Not applicable.
